# Cerebrospinal Fluid Dendritic Cells Infiltrate the Brain Parenchyma and Target the Cervical Lymph Nodes under Neuroinflammatory Conditions

**DOI:** 10.1371/journal.pone.0003321

**Published:** 2008-10-02

**Authors:** Eric Hatterer, Monique Touret, Marie-Françoise Belin, Jérôme Honnorat, Serge Nataf

**Affiliations:** 1 INSERM, U842, Lyon, France; 2 Université de Lyon, Lyon1, UMR-S842, Lyon, France; University Paris Sud, France

## Abstract

**Background:**

In many neuroinflammatory diseases, dendritic cells (DCs) accumulate in several compartments of the central nervous system (CNS), including the cerebrospinal fluid (CSF). Myeloid DCs invading the inflamed CNS are thus thought to play a major role in the initiation and perpetuation of CNS-targeted autoimmune responses. We previously reported that, in normal rats, DCs injected intra-CSF migrated outside the CNS and reached the B-cell zone of cervical lymph nodes. However, there is yet no information on the migratory behavior of CSF-circulating DCs under neuroinflammatory conditions.

**Methodology/Principal Findings:**

To address this issue, we performed *in vivo* transfer experiments in rats suffering from experimental autoimmune encephalomyelitis (EAE), a model of multiple sclerosis. EAE or control rats were injected intra-CSF with bone marrow-derived myeloid DCs labeled with the fluorescent marker carboxyfluorescein diacetate succinimidyl ester (CFSE). In parallel experiments, fluorescent microspheres were injected intra-CSF to EAE rats in order to track endogenous antigen-presenting cells (APCs). Animals were then sacrificed on day 1 or 8 post-injection and their brain and peripheral lymph nodes were assessed for the presence of microspheres^+^ APCs or CFSE^+^ DCs by immunohistology and/or FACS analysis. Data showed that in EAE rats, DCs injected intra-CSF substantially infiltrated several compartments of the inflamed CNS, including the periventricular demyelinating lesions. We also found that in EAE rats, as compared to controls, a larger number of intra-CSF injected DCs reached the cervical lymph nodes. This migratory behavior was accompanied by an accentuation of EAE clinical signs and an increased systemic antibody response against myelin oligodendrocyte glycoprotein, a major immunogenic myelin antigen.

**Conclusions/Significance:**

Altogether, these results indicate that CSF-circulating DCs are able to both survey the inflamed brain and to reach the cervical lymph nodes. In EAE and maybe multiple sclerosis, CSF-circulating DCs may thus support the immune responses that develop within and outside the inflamed CNS.

## Introduction

DCs are the most powefull antigen presenting-cells of the immune system. They sequentially capture antigens in inflamed tissues, reach the lymphatic vessels, migrate toward lymphoid organs and induce the antigen-specific proliferation of T-cells [1], [2]. However, this functional scheme does not apply to CNS for the following reasons: i) in contrast with all other tissues, there is no DCs residing in the CNS parenchyma, ii) the so-called “blood-brain barrier” considerably limits the penetration of blood-circulating immune cells, including DCs and their precursors, into the CNS parenchyma; iii) the CNS is devoided of lymphatic vessels. Nevertheless, despite these limitations, DCs were shown to infiltrate several compartments of the CNS under neuroinflammatory conditions. These intra-CNS compartments communicate with each others and comprises: the CSF [3], [4], the meninges [5], [6], the perivascular spaces [5], [6] and the CNS parenchyma [6], [7]. Due to the lack of intra-CNS lymphatic vessels, the question whether and how DCs migrate from the inflamed CNS to lymphoid organs is still controversial. Previous studies performed in normal rats or mice showed that DCs are able to migrate from brain to cervical lymph nodes (CLNs) and to elicit a systemic immune response [8], [9]. Also, we reported that in normal rats, DCs injected into the cerebrospinal fluid reached the CLNs while DCs injected into the brain parenchyma stayed confined to the CNS [10]. However, as these experiments were performed in normal rats, one cannot conclude on the actual behavior of CNS-infiltrating DCs under neuroinflammatory conditions. In the present paper, we specifically assessed the migratory behavior and functions of CSF-circulating DCs (CSF DCs) in a rat model of multiple sclerosis, the most common autoimmune disorder of the CNS. Two complementary experimental procedures were followed: i) in a first set of experiments, we tracked endogenous CSF-circulating antigen-presenting cells (APCs) by injecting fluorescent microspheres into the CSF of rats induced for EAE; ii) in a second set of experiments, bone marrow-derived myeloid DCs were labeled with the fluorescent marker carboxyfluorescein diacetate succinimidyl ester (CFSE) then injected into the CSF of EAE rats, at the clinical peak of the disease, or control rats. The brains and peripheral lymph nodes of injected animals were then examined by immunohistological methods or FACS analysis on day 1 or 8 post-injection. Data showed that in EAE rats, CSF DCs substantially infiltrated the inflamed brain. Moreover, CSF DCs reached the cervical lymph nodes and enhanced the systemic humoral response against myelin oligodendrocyte glycoprotein (MOG), a major immunogenic myelin antigen,

## Results

### Intra-CNS migration of CSF DCs in EAE versus control rats

An immunohistological examination of the CNS was performed in: i) EAE rats injected intra-CSF with microspheres (n = 14) and sacrificed on day 1 (n = 8) or 8 (n = 6) post-injection; ii) EAE rats injected intra-CSF with CFSE-labeled DCs (n = 18) and sacrificed on day 1 (n = 8) or 8 (n = 10) post-injection; iii) control rats injected intra-CSF with CFSE-labeled DCs and sacrificed on day 1 post-injection (n = 4) or 8 post-injection (n = 3). In EAE rats, all intra-CSF injections were performed at the clinical peack of disease, on day 12 post-immunization.

### (i) Migration of CSF DCs in the periventricular parenchyma

**Figure 1:**
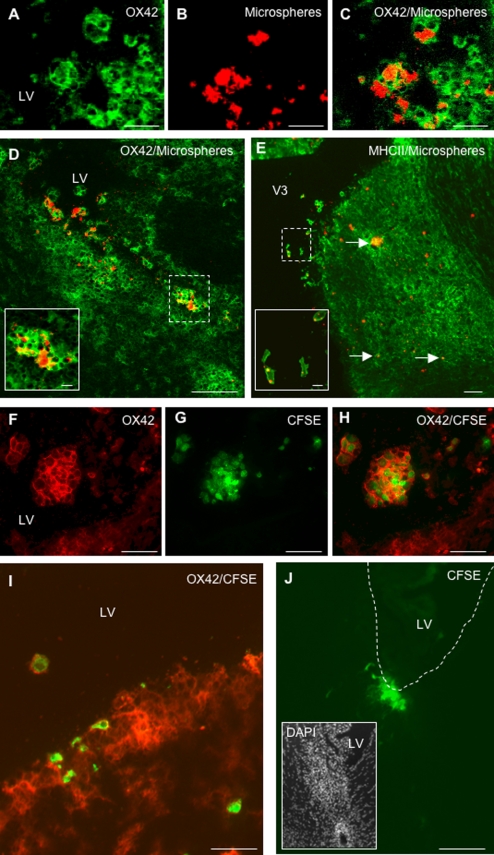
CSF-circulating DCs infiltrate the periventricular parenchyma. Fluorescent microspheres or DCs labeled with the cytoplasmic fluorescent marker CFSE were injected into the left lateral ventricle of EAE rats (n = 32) at the clinical peak of disease (day 12 post-immunization). EAE rats injected with fluorescent microspheres (n = 14) or CFSE-labeled DCs (n = 18) were then sacrificed on day 1 post-injection (n = 16) or 8 post-injection (n = 16). An immunohistological analysis of brains was then performed using antibodies directed against CD11b/CD11c (OX42) or MHC class II molecules. A–C: OX42^+^ cells (green) harboring intracytoplasmic fluorescent microspheres (red) are observed in the lumen of the injected lateral ventricle, on day 1 post-injection. D: Microspheres^+^/OX42^+^ cells localize in the periventricular parenchyma of the injected ventricle, on day 1 post-injection. Insert (solid square) shows a high magnification view of microspheres^+^/OX42^+^ cells infiltrating a periventricular inflammatory lesion (dashed square). E: On day 1 post-injection, MHC class II^+^ cells (green) harboring intracytoplasmic fluorescent microspheres (red) are detectable in the lumen of the third ventricle (dashed square) and in a large periventricular area infiltrated with MHC class II^+^ cells. Insert (solid square) shows a high magnification view of microspheres^+^/MHC class II^+^ cells that localize in the lumen of the third ventricle (dashed square). Arrows indicate microspheres^+^/MHC class II^+^ cells in the periventricular parenchyma. F–H: CFSE^+^ DCs (green) that express OX42 (red) are detected in the lumen of the injected lateral ventricle, on day 1 post-injection. I: A periventricular area bordering the injected lateral ventricle is infiltrated by OX42^+^ cells and contains OX42^+^/CFSE^+^ DCs, on day 1 post-injection. J: On day 8 post-injection, CFSE^+^ DCs are observed in a periventricular inflammatory lesion, adjacent to the injected lateral ventricle. LV: lateral ventricle, V3: third ventricle. Scale bars: 100 µm (B–J), 50 µm (A–C, E–I), 10 µm (inserts in panel D and E).

**Figure 2:**
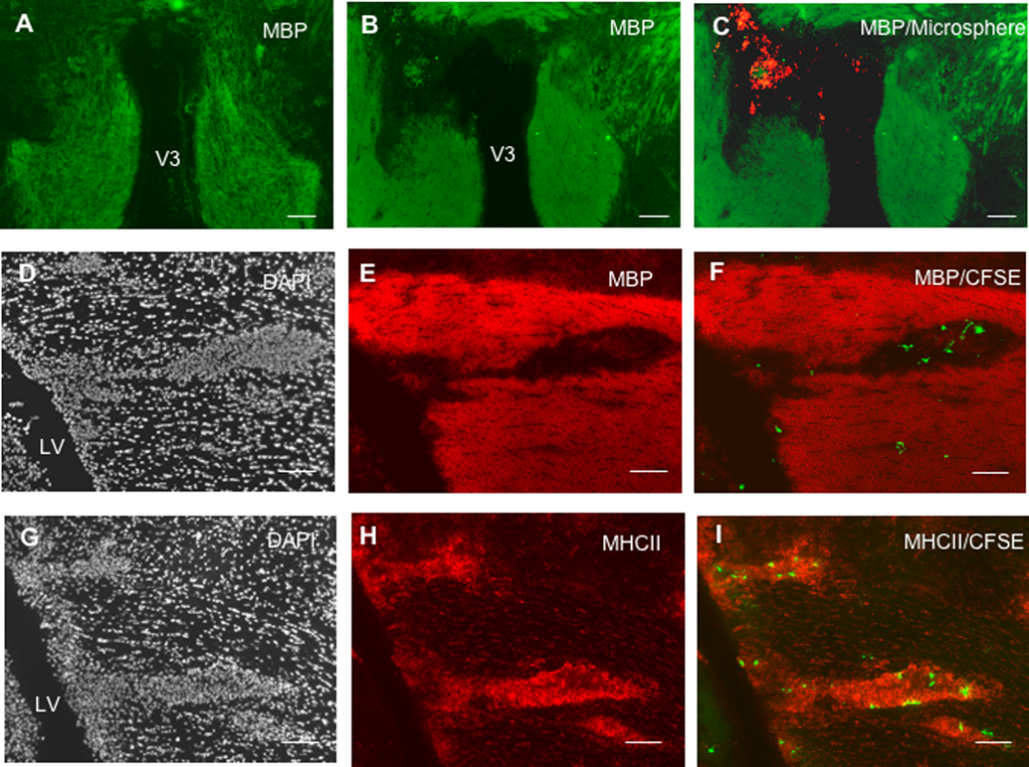
CSF-circulating DCs penetrate periventricular demyelinating lesion. Fluorescent microspheres or DCs labeled with the cytoplasmic fluorescent marker CFSE were injected into the left lateral ventricle of EAE rats (n = 32) at the clinical peak of disease (day 12 post-immunization). EAE rats injected with fluorescent microspheres (n = 14) or CFSE-labeled DCs (n = 18) were then sacrificed on day 1 post-injection (n = 16) or 8 post-injection (n = 16). An immunohistological analysis of brains obtained from injected EAE rats or control healthy rats (n = 4) was then performed using antibodies directed against MHC class II molecules and/or myelin basic protein (MBP). A–C: In a normal rat (A), immunostaining of MBP (green) is homogenous and symmetric in the periventricular parenchyma adjacent to the third ventricle. In contrast, a large periventricular demyelinated area, adjacent to the third ventricle, is observed in an EAE rat injected intra-CSF with microspheres and sacrificed on day 8 post-injection (B). This demyelinating lesion is filled with microspheres^+^ cells (C). D–F: On day 1 post-injection, an area of periventricular infiltration adjacent to the injected lateral ventricle (D) is demyelinated (E) and contains CFSE^+^ DCs (F). G–I: On day 1 post-injection, periventricular parenchymal infiltrates (G) adjacent to the injected lateral ventricle are formed by MHC class II^+^ cells (H) and contain MHC class II^+^/CFSE^+^ DCs (I). LV: lateral ventricle, V3: third ventricle. Scale bars: 50 µm (A–I).

**Figure 3:**
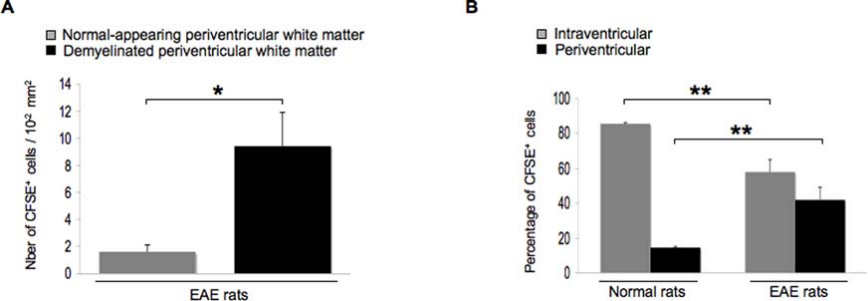
CFSE^+^ DCs specifically target the periventricular demyelinating lesions. DCs labeled with the cytoplasmic fluorescent marker CFSE were injected into the left lateral ventricle of healthy control rats or EAE rats at the clinical peak of disease (day 12 post-immunization). Rats were then sacrificed on day 1 post-injection (EAE rats: n = 4, normal rats: n = 4) and an immunohistological analysis was performed on brain sections crossing the injected lateral ventricle. At least 3 to 5 sections per animal were examined. A: In brain sections from EAE rats, an immunostaining of the myelin basic protein (MBP) was performed and the number of CFSE^+^ cells/10^−2^ mm^2^ was counted in demyelinated vs normal-appearing periventricular white matter, as described in the Materials and Methods section. Data show that the mean number of CFSE^+^ cells was more than 4 times higher in periventricular demyelinating lesions than in normal-appearing periventricular white matter (9.4+/−2.5 cells/10^−2^ mm^2^ vs 1.6+/−0.5 cells/10^−2^ mm^2^ in demyelinated vs normal-apprearing periventricular white matter respectively, p = 0.02, Mann and Whitney test). B: Intraventricular vs periventricular CFSE^+^ cells were counted in brain sections obtained from injected EAE rats or injected control rats. Data are presented as percentages of intraventricular vs periventricular cells. Results show that in normal rats, 14.5+/−0.6% cells localized in the periventricular parenchyma, while, in EAE rats, more than 40% of the injected cells localized in the periventricular parenchyma (42+/−7%, p = 0.008, Mann and Whitney test). Conversely, 85.5+/−0.6% CFSE^+^ cells localized in the intraventricular lumen of normal rats while less than 60% of the injected cells localized in the intraventricular lumen of EAE rats (58+/−7%, p = 0.008, Mann and Whitney test). *: p<0.05, **: p<0.01.

In EAE rats injected with microspheres and sacrificed on day 1 post-injection (n = 8), phagocytic cells having engulfed fluorescent microspheres (microspheres^+^ cells) were detected as free-floating cells in the injected lateral ventricle (Fig 1A–D) as well as in the third ventricle (Fig 1E) and in the fourth ventricle (data not shown). These microspheres^+^ cells expressed OX42 (CD11b/c) and MHC class II molecules (Fig 1A–C). Also, microspheres^+^ cells harboring the same phenotype were evidenced in periventricular inflammatory lesions (Fig 1D–E). Similarly, in EAE rats injected with CFSE-labeled DCs and sacrificed on day 1 post-injection (n = 8), CFSE^+^/OX42^+^ cells were detected in the ventricular CSF compartment (Fig 1F–H) and in the periventricular parenchyma (Fig 1I). A similar distribution of CFSE+/OX42+ cells was observed in EAE rats injected with CFSE-labeled DCs and sacrificed on day 8 post-injection (n = 10) (Fig 1J). When performing an immunostaining of the myelin basic protein (MBP)(Fig 2), we found that microspheres^+^ cells (Fig 2A–C) or CFSE^+^/MHC class II^+^ DCs (Fig 2D–I) could be evidenced within periventricular demyelinating lesions, on day 8 (Fig 2A–C) or 1 post-injection (Fig 2D–I). Such a migratory behavior was specific as the mean number of CFSE^+^ DCs was more than 4 times higher in periventricular demyelinating lesions than in normal-appearing periventricular white matter (9.4+/−2.5 cells/10^−2^ mm^2^ vs 1.6+/−0.5 cells/10^−2^ mm^2^ in demyelinated vs normal-appearing periventricular white matter respectively, p = 0.02, Mann and Whitney test)(Fig 3A). Interestingly, some of the microspheres^+^ cells that localized in these periventricular demyelinating lesions displayed an intracytoplasmic MBP staining, suggesting they had ingested MBP-containing myelin debris (Data supplement S1). Finally, to further determine whether the intra-CNS migration of CSF DCs is conditionned by the neuroinflammatory environment, we performed parallel experiments in which CFSE^+^ DCs were injected into the CSF of normal rats (n = 4). Rats were sacrificed on day 1 post-injection and serial sections of the injected lateral ventricle were examined. CFSE^+^ DCs were then counted in order to establish the respective percentages of intraventricular vs periventricular CFSE^+^ DCs on day 1 following injection (Fig 3B). The same procedure was applied to EAE rats injected with CFSE-labeled DCs and sacrificed on day 1 post-injection (n = 7). While in normal rats, 14.5+/−0.6% cells localized in the periventricular parenchyma, we found that in EAE rats more than 40% of the injected cells localized in the periventricular parenchyma (42+/−7%, p = 0.008, Mann and Whitney test).

Altogether these data show that in EAE rats as compared to controls, CSF DCs present an increased ability to penetrate the periventricular parenchyma and, in particular, the demyelinating lesions that are adjacent to ventricles.

### (ii) Migration of CSF DCs along the deep penetrating meninges

**Figure 4:**
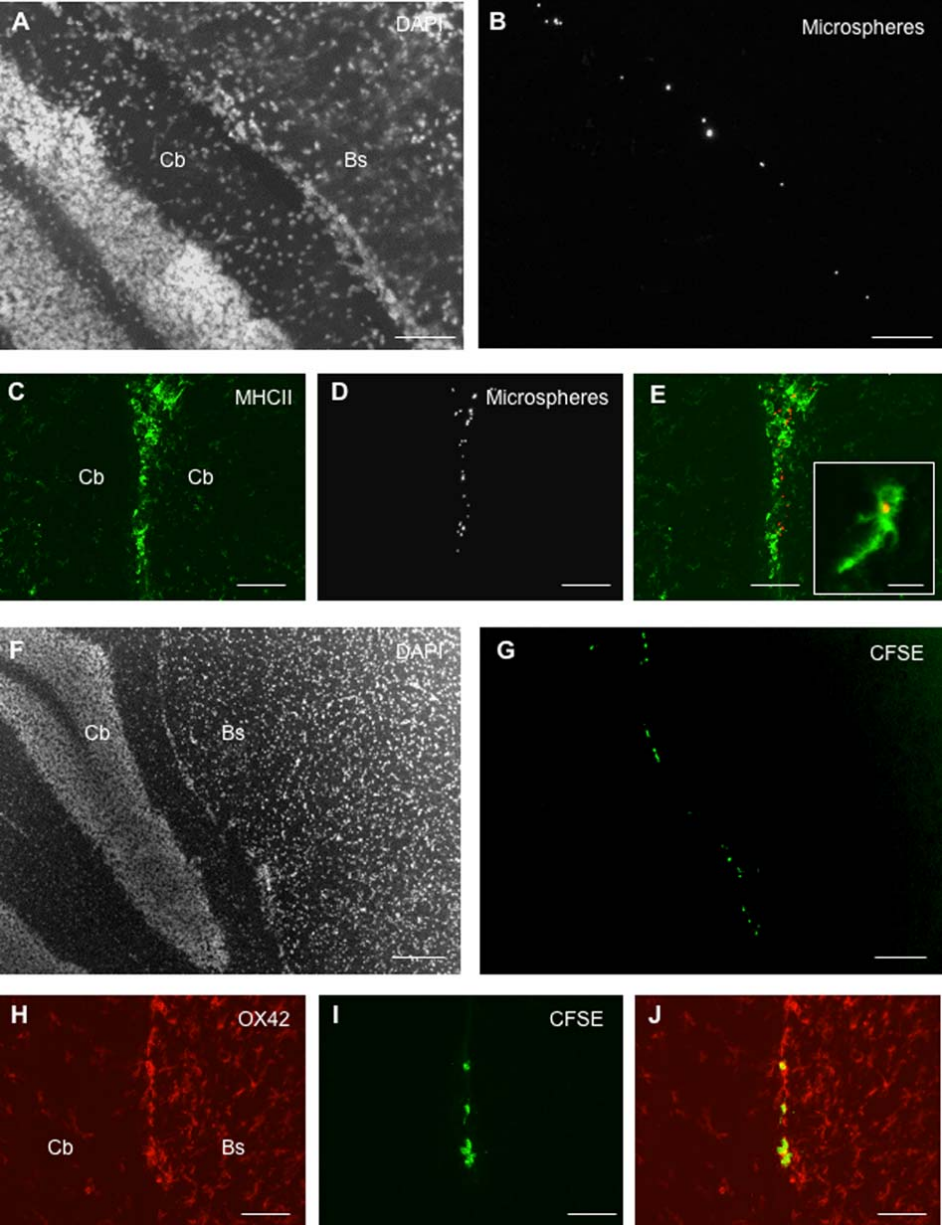
CSF-circulating DCs migrate along the deep penetrating meninges. Fluorescent microspheres or DCs labeled with the cytoplasmic fluorescent marker CFSE were injected into the left lateral ventricle of EAE rats (n = 32) at the clinical peak of disease (day 12 post-immunization). EAE rats injected with fluorescent microspheres (n = 14) or CFSE-labeled DCs (n = 18) were then sacrificed on day 1 post-injection (n = 16) and an immunohistological analysis of brains was performed using antibodies directed against CD11b/CD11c (OX42) or MHC class II molecules. Nuclei were counterstained with the fluorescent nuclear dye DAPI. A, B: Counterstaining of nuclei with DAPI coloration (A) shows that microspheres^+^ cells (visualized as white spots in B) localize in the deep penetrating meninges lining the inner parts of the brainstem (Bs) and cerebllum (Cb). C–E: Microspheres^+^ cells (white in D, red in E) expressing MHC class II molecules (green in C and E) are observed in the deep penetrating meninges covering the cerebellar convolutions. Insert in E shows a high magnification view of a ramified microsphere^+^/MHC class II^+^ cell observed in the penetrating pia matter. F, G: Counterstaining of nuclei with DAPI coloration (F) shows that CFSE^+^ cells (G) localize along the pia matter lining the inner parts of the brainstem (Bs) and cerebellum (Cb). H–J: An OX42^+^ infiltrate is observed in the deep penetrating meninges covering the inner parts of the brainstem (Bs) and cerebellum (Cb) (H). These infiltrating cells comprise CFSE^+^ cells (I) that express OX42 (J). Scale bars: 100 µm (F–G), 50 µm (A–E, H–J), 10 µm (insert in panel E).

On day 1 or 8 following injection, numerous microspheres^+^ cells were evidenced in the superficial meninges covering the outer surface of the brain (data not shown). However, microspheres^+^ cells were also frequently detected in the deep penetrating meninges (Fig 4). In particular, microspheres+ cells localized along the pia matter covering the molecular layers of the cerebellum (Fig 4A–B) or lining the inner surface of the brainstem (data not shown). These microspheres+ cells expressed MHC class II molecules (Fig 4C–E) and OX42 (data not shown). Similarly, on day 1 post-injection, numerous CFSE^+^ DCs were evidenced in the deep penetrating meninges (Fig 4C, D). As observed for microspheres^+^ cells, CFSE^+^ DCs were frequently detected in the meninges covering the molecular layers of the cerebellum (data not shown) or in the meninges lining the inner surface of the brainstem (Fig 4F–G). These cells expressed MHC class II molecules and OX42 (Fig 4H–J and data not shown). However, on day 8 following injection, only few CFSE^+^ cells were still detectable in the deep penetrating meninges (data not shown). Interestingly, when CSFE^+^ DCs were injected into the CSF of normal rats, we could not detect CFSE^+^ cells in the deep penetrating meninges (Table 1).

**Table 1:** Semi-quantitative distribution of CSF-injected CFSE^+^ DCs in the CNS of normal or EAE rats.

CNS compartment	Normal rat	EAE rat
***Perivascular***	+/−	+
***Intraventricular***		
Free-floating	+++	+++
Adherent to choroid plexuses	+++	+++
***Periventricular***		
LV	+ (SVZ)	+++
V3	+/−	++
V4	+/−	++
***Brain meninges***		
Superficial	+++	+++
Penetrating	−	++
***Cerebellum meninges***		
Superficial	+++	+++
Penetrating	−	+++
***Cerebellum parenchyma***	−	+
***Brain stem parenchyma***	−	+

In parallel experiments, DCs labeled with the cytoplasmic fluorescent marker CFSE were injected intra-CSF to normal rats (n = 7) or EAE rats (n = 8). Serial brain sections crossing different brain areas (anterior brain, midbrain, brain stem and cerebellum) were then assessed for the presence of CFSE^+^ cells, on day 1 post-injection (EAE rats: n = 4, normal rats: n = 4). A semi-quantitative analysis was performed as follows: −: none; +/−: occasional cells; +: 2 to 5 cells per section; ++: 5 to 10 cells per section; +++: >10 cells per section. LV: lateral ventricle, V3: third ventricle, V4: fourth ventricle, SVZ: subventricular zone.

### (iii) Migration of CSF DCs in the brain parenchyma and perivascular spaces

**Figure 5:**
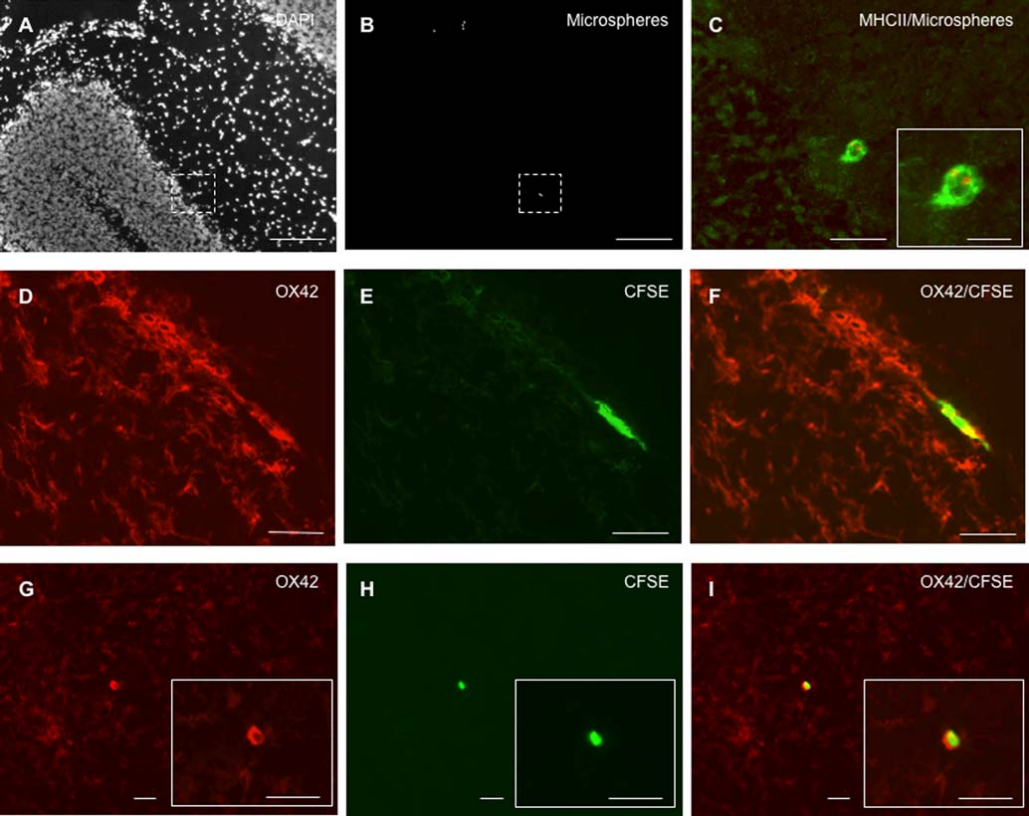
CSF-circulating DCs infiltrate the brain parenchyma. Fluorescent microspheres or DCs labeled with the cytoplasmic fluorescent marker CFSE were injected into the left lateral ventricle of EAE rats (n = 32) at the clinical peak of disease (day 12 post-immunization). EAE rats injected with fluorescent microspheres (n = 14) or CFSE-labeled DCs (n = 18) were then sacrificed on day 1 post-injection (n = 16) and their brains examined by immunohistology using antibodies directed against CD11b/CD11c (OX42) or MHC class II molecules. Nuclei were counterstained with the fluorescent nuclear dye DAPI. A–C: Visualization of nuclei with DAPI coloration (A) allows the localization of microspheres (white in B, red in C) to be determined (dashed squares in A and B). Microspheres are detected in the molecular layer of the cerebellum and localize in the cytoplasm of a MHC class II^+^ cell (C). Insert in C shows a high magnification view of this microspheres^+^/MHC class II^+^ cell. DAPI coloration (A) and MHC class II staining (C) shows that there is no detectable inflammatory infiltrate in this area of the cerebellum. D–F: In the brainstem, an intraparenchymal inflammatory infiltrate is formed by OX42^+^ cells (D) and contains a CFSE^+^ cell (E) that expresses OX42 (F). G–I: In the brainstem, an area of diffuse infiltration with OX42^+^ cells (G) contains a CFSE^+^ cell (H) that expresses OX42 (I). Inserts in G, H and I show high magnification views of this CFSE^+^/OX42^+^ cell. Scale bars: 100 µm (A, B), 50 µm (C–I), 10 µm (insert in panel C).

**Figure 6:**
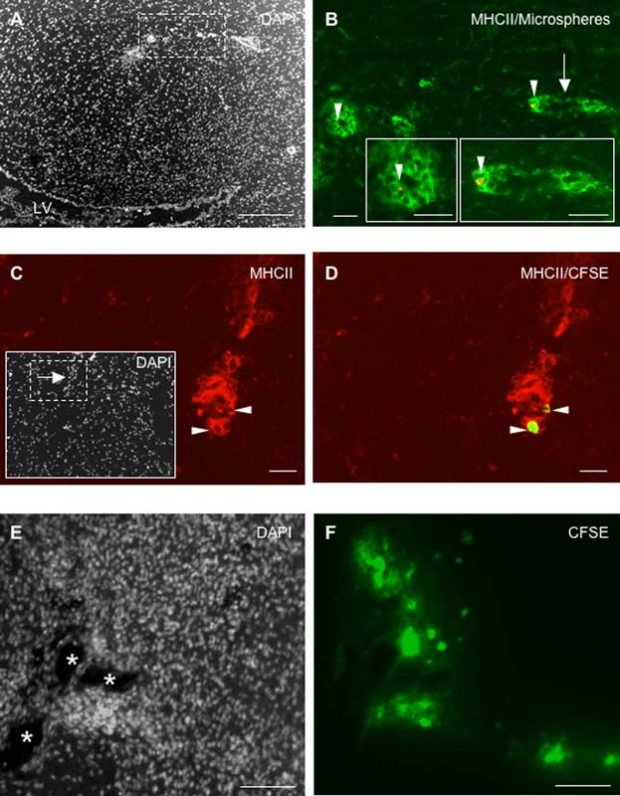
CSF-circulating DCs migrate toward intraparenchymal perivascular infiltrates. Fluorescent microspheres or DCs labeled with the cytoplasmic fluorescent marker CFSE were injected into the left lateral ventricle of EAE rats (n = 32) at the clinical peak of disease (day 12 post-immunization). EAE rats injected with fluorescent microspheres (n = 14) or CFSE-labeled DCs (n = 18) were then sacrificed on day 1 post-injection (n = 16) or 8 post-injection (n = 16). Their brains were then examined by immunohistology using antibodies directed against MHC class II molecules. Nuclei were counterstained with the fluorescent nuclear dye DAPI. A, B: Two cuffed vessels (dashed square in A) are observed in the brain parenchyma, distant away from the injected lateral ventricle, on day 1 post-injection. These perivascular infiltrates contain microspheres^+^/MHC class II^+^ cells (arrow heads in B). One of the cuffed vessels presents a venule-like morphology (arrow). Inserts in B show high magnification views of these microspheres^+^/MHC class II^+^ cells. C, D: In the brainstem, a perivascular infiltrate is formed by MHC class II^+^ cells (C) and contains CFSE^+^/MHC class II^+^ cells (arrow heads in C and D) on day 1 post-injection. Insert in C shows a DAPI coloration of the brainstem area where this cuffed vessel localizes (arrow in dashed square). E, F: In the hippocampus, on day 8 post-injection, three cuffed vessels harboring a venule-like morphology (white stars in E) are surrounded by CFSE^+^ cells (F). Scale bars: 100 µm (A), 50 µm (B, E, F), 20 µm (C, D).

On day 1 or 8 post-injection, microspheres^+^/MHC class II^+^ cells as well as CFSE^+^ DCs localized in parenchymal areas, distant away from the site of injection (Fig 5 and 6). In some cases, these cells appeared to infiltrate a non-inflamed parenchymal area as judged by the lack of infiltrating MHC class II^+^ or OX42^+^ inflammatory cells (Fig 5A–C). In other cases, CSF-derived DCs were clearly localized within parenchymal inflammatory foci. (Fig 5D–I). Interestingly, microspheres^+^/MHC class II^+^ cells or CFSE^+^ DCs were demonstrated in perivascular infiltrates (Fig 6). Indeed, as early as day 1 post-injection, microspheres^+^/MHC class II^+^ cells or CFSE^+^/MHC class II^+^ DCs were detected in close vicinity with the vascular wall of cuffed vessels (Fig 6A–D) including small caliber venules (Fig 6B, arrow). A similar migratory pattern was observed on day 8 post-injection (Fig 6E–F). In comparison, only a few CFSE^+^ cells were detected in the perivascular spaces of normal rats injected with CFSE^+^ DCs (Table 1).

### Migration of CSF DCs in the cervical lymph nodes

**Figure 7:**
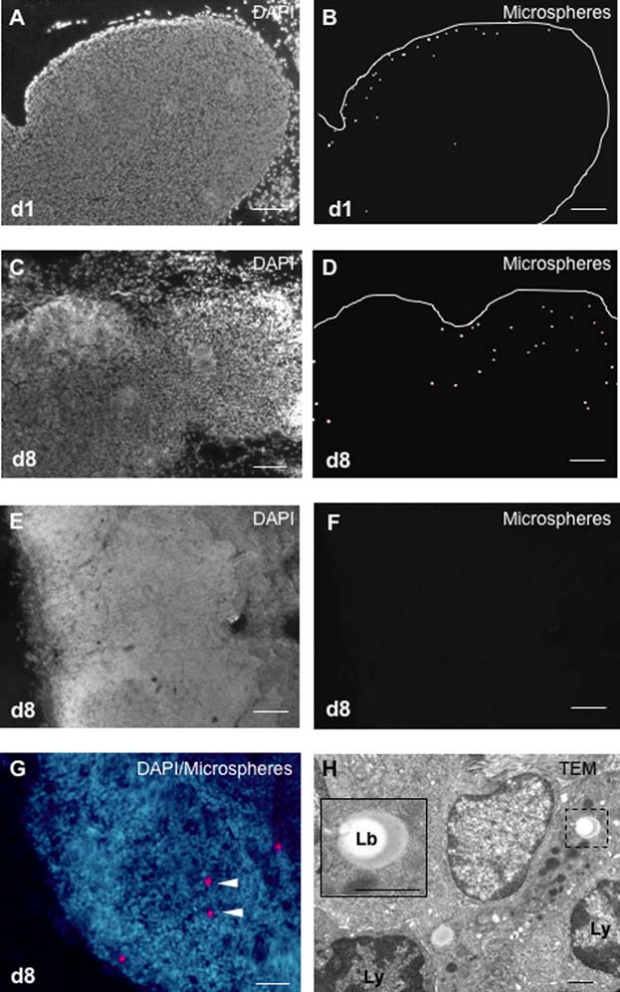
CSF-derived microspheres^+^ cells target the B-cell zone of CLNs. Fluorescent microspheres were injected into the left lateral ventricle of EAE rats (n = 14) at the clinical peak of disease (day 12 post-immunization). EAE rats were then sacrificed on day 1 post-injection (n = 8) or 8 post-injection (n = 6) and their cervical lymph nodes and axillary lymph nodes were examined by histological methods. Nuclei were counterstained with the fluorescent nuclear dye DAPI. A–D: In the cervical lymph nodes, on day 1 (d1)(A, B) or 8 (d8)(C, D) post-injection, microspheres^+^ cells (visualized as white spots) localize preferentially in the cortical, B-cell rich zone. E, F: On day 8 post-injection (d8), there is no detectable microspheres^+^ cell in the axillary lymph nodes. G: On day 8 post-injection (d8), in the cervical lymph nodes, microspheres^+^ cells (arrow heads)(red spots) are observed in a B-cell follicle (arrow heads). H: On day 8 post-injection, analysis of cervical lymph nodes by transmission electron microscopy (TEM) shows, in the cortical zone, a phagocytic cell containing a latex bead (Lb, dashed square) and localizing in close contact with lymphocytes (Ly). A high magnification view of this engulfed latex bead (Lb) is shown in the insert (solid square). Scale bars: 100 µm (A–F), 50 µm (G), 1 µm (H).

A histological examination of the cervical lymph nodes and axillary lymph nodes was performed in EAE rats injected intra-CSF with microspheres (n = 14) and sacrificed on day 1 (n = 8) or 8 (n = 6) post-injection. On day 1 or 8 following injection, fluorescent microspheres were evidenced in the cervical lymph nodes (CLNs) (Fig 7A–B) but not in the axillary lymph nodes (Fig 7E–F) of EAE rats. Microspheres preferentially localized in the B-cell area of CLNs and could be demonstrated within the germinal centers of B-cell follicles (Fig 7G). In order to formally confirm that microspheres had not been passively transported in the lymph flux, an ultrastructural analysis of CLNs was performed. This allowed microspheres to be demonstrated in the cytoplasm of phagocytic cells that localized in the B-cell areas of CLNs (Fig 7H).

**Figure 8:**
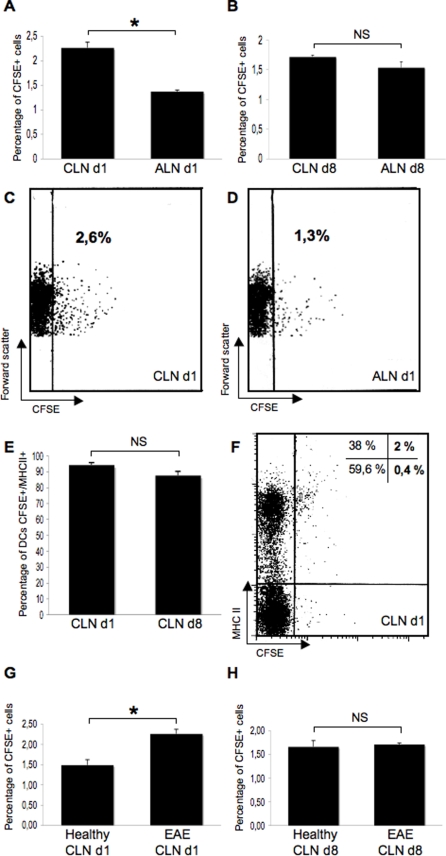
CSF-circulating DCs target the CLNs. In parallel experiments, DCs labeled with the cytoplasmic fluorescent marker CFSE were injected into the left lateral ventricle of control rats (n = 7) or EAE rats (n = 8) at the clinical peak of disease (day 12 post-immunization). Rats were then sacrificed on day 1 (d1) or 8 (d8) following injections. The cervical lymph nodes (CLNs) and axillary lymph nodes (ALN) were assessed by FACS analysis for the presence of CFSE^+^ cells. The level of autofluorescence was established on cells obtained from the CLNs or ALNs of non-injected EAE rats (n = 4) or control rats (n = 3). A–D: In injected EAE rats sacrificed on day 1 post-injection (n = 4), we found that 2.26+/−0.11% CFSE^+^ cells could be detected in the CLNs as compared to 1.37+/−0.03% in the axillary lymph nodes (p = 0.0202, Mann and Whitney test)(A). However, in injected EAE rats sacrificed on day 8 post-injection (n = 4), the percentage of CFSE^+^ cells was not statistically different between the CLNs and the ALNs (B). Pannels C and D show representative dot plots obtained from the analysis of injected EAE rats sacrificed on day 1 post-injection. E–F: In the CLNs of injected EAE rats, a great majority of CFSE^+^ cells express MHC class II molecules, on day 1 post-injection (94.25+/−3.3%) or 8 post-injection (87.65+/−5.2%). A representative dot plot is shown in F. G–H: When comparing injected EAE rats to injected control rats, data showed that on day 1 post-injection, a greater pourcentage of CSFE^+^ cells was detectable in the CLNs of injected EAE rats as compared to injected control rats (2.26+/−0.11% vs 1.49+/−0.13% in injected EAE and injected control rats respectively, p = 0.0339, Mann and Whitney test)(G). This difference did not reach significance on day 8 post-injection (1.71+/−0.03% vs 1.66+/−0.13% in injected EAE and injected control rats respectively) (H). *: p<0.05, NS: not significant.

To gain quantitative insights on the migration of CSF DCs toward the CLNs, a FACS analysis was performed on the lymph nodes of EAE rats (n = 8) or control rats (n = 7) injected intra-CSF with CFSE^+^ DCs (Fig 8). In injected EAE rats sacrificed on day 1 post-injection (n = 4), we found that 2.26+/−0,11% CFSE^+^ cells could be detected in the CLNs as compared to 1.37+/−0,03% in the axillary lymph nodes (p = 0.02, Mann and Whitney test)(Fig 8A, C–D). However, in injected EAE rats sacrificed on day 8 post-injection (n = 4), no statistical difference was observed between the CLNs and the axillary lymph nodes regarding the percentage of CFSE^+^ cells (Fig 8B). It has to be noticed that a dilution effect may have partly biased these results as cellularity increases in the CLNs during the course of EAE (data not shown). It is also noteworthy that in the CLNs, a majority of CFSE^+^ cells were MHC class II^+^ on day 1 or 8 post-injection (Fig 8E–F). This suggests that in the CLNs, the phenotype of injected cells is stable over time, at least regarding the expression of MHC class II molecules.

The presence of CFSE^+^ cells, as assessed by FACS analysis, was then compared in the CLNs of injected EAE rats (n = 8) vs injected control rats (n = 7) on day 1 or 8 post-injection. Data showed that on day 1 post-injection, a greater pourcentage of CSFE^+^ cells was detectable in the CLNs of injected EAE rats (n = 4) as compared to injected control rats (n = 3) (2.26+/−0.11% vs 1.49+/−0.13% in EAE and control rats respectively, p = 0.03, Mann and Whitney test) (Fig 8G). Again, this difference did not reach significance on day 8 post-injection (Fig 8H).

Overall, these results show that CSF-circulating APCs rapidly and specifically target the CLNs under neuroinflammatory conditions. This process is, at least partly, dependent on the immune processes that take place in the CNS and the CLNs.

### Effects of intra-CSF injection of DCs on EAE outcome

**Figure 9:**
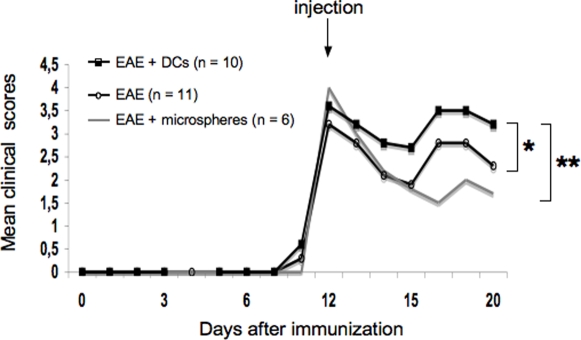
DCs injected intra-CSF aggravates EAE clinical signs. The clinical course of EAE was compared between control EAE rats (n = 11), EAE rats injected intra-CSF with microspheres (n = 6) and EAE rats injected intra-CSF with CFSE-labeled DCs (n = 10). Data show that in the time period between day 12 (intra-CSF injections) and day 20 (sacrifice), EAE rats injected with DCs presented higher clinical scores (cumulative clinical score: 22.25+/−1.4) than EAE control rats (cumulative clinical score: 17.45+/−7.09, p = 0.0409 as compared to EAE rats injected with DCs, Student's *t* test) or EAE rats injected with microspheres (cumulative clinical score: 14.83+/−1.6; p = 0.0020 as compared to EAE rats injected with DCs, Student's *t* test). In contrast, the cumulative clinical scores observed in EAE control rats and EAE rats injected with microspheres were not statistically different. *: p<0.05, **: p<0.01.

**Figure 10:**
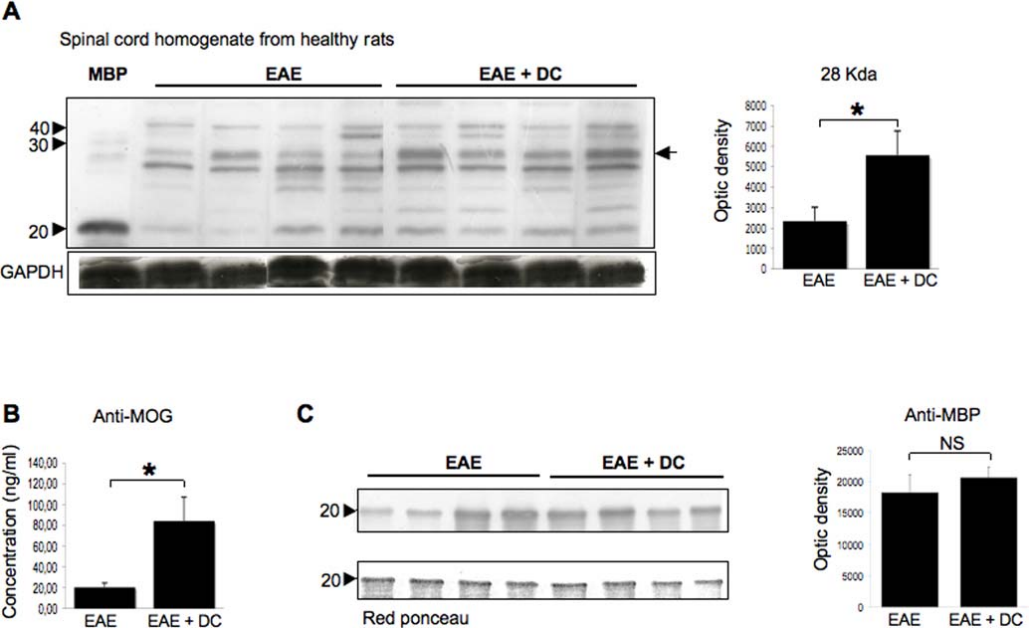
DCs injected intra-CSF stimulates the antibody response against myelin oligodendrocyte glycoprotein. On day 20 post-immunization, blood samples were withdrawn from control EAE rats (n = 4) or EAE rats that had been injected intra-CSF with DCs on day 12 post-immunization (n = 4). Sera were then assessed by Western blot analysis or ELISA for the presence of antibodies directed against CNS antigens. A. The serum antibody repertoire against whole spinal cord homogenate (obtained from healthy rats) was profiled by Western blot analysis (left panel) followed by measures of optical densities (right panel). Data showed that, as compared to control EAE rats, sera from injected EAE rats contained higher concentrations of antibodies directed against a 28 Kda protein, distinct from myelin basic protein (MBP, a major immunogenic myelin antigen) (optic density: 5583.9+/−1161.5 vs 2343+/−677 in EAE+DC and EAE rats respectively; p = 0.0433, non parametric Mann and Whitney test). B. As myelin oligodendrocyte glycoprotein (MOG, another major immunogenic myelin antigen) is a 28 Kda protein, we performed ELISA experiments allowing serum antibodies against MOG peptide 35–55 to be measured. Data showed that higher concentrations of anti-MOG antibodies were detectable in injected EAE rats (EAE+DCs) as compared to control EAE rats (EAE) (84.23+/−22.8 ng/ml vs 20.53+/−4.1 ng/ml in EAE+DCs vs EAE rats respectively, p = 0.0273, non parametric Mann and Whitney test). C. Western blot experiments (left panel) followed by quantitative analysis of optical densities (right panel) showed that the serum antibody response against purified myelin basic protein (MBP) was not significantly different in EAE injected rats (EAE+DCs) as compared to control EAE rats (EAE) (optic density: 20572+/−1683.93 vs 18209+/−2932.34 in EAE+DCs vs EAE rats respectively, p: not significant). Membranes were stained with Red ponceau (lower left panel) to ensure that similar amounts of purified MBP had been loaded in the different lanes. *: p<0.05.

The clinical course of EAE was compared between control EAE rats, EAE rats injected intra-CSF with microspheres and EAE rats injected intra-CSF with CFSE-labelled DCs As shown in Figure 9, we observed that in the time period between day 12 (intra-CSF injections) and day 20 (sacrifice), EAE rats injected with DCs (n = 10) presented higher clinical scores (cumulative clinical score: 22.25+/−1.4) than EAE control rats (n = 11, cumulative clinical score: 17.45+/−7.09, p = 0.04 as compared to EAE rats injected with DCs, Student's *t* test) or EAE rats injected with microspheres (n = 6, cumulative clinical score: 14.83+/−1.6; p = 0.002 as compared to EAE rats injected with DCs, Student's *t* test). We then attempted to determine whether intra-CSF injections of DCs had induced an increased peripheral immune response against CNS antigens. As microspheres^+^ CSF-derived APCs targeted the B-cell follicles of CLNs, we focused our analysis on the B-cell response against CNS antigens. On day 20 post-immunization, blood samples were withdrawn from control EAE rats (n = 4) or EAE rats that had been injected intra-CSF with DCs on day 12 post-immunization (n = 4). Sera were then assessed by Western blot or ELISA for the presence of antibodies directed against CNS antigens. We first profiled the serum antibody repertoire against whole spinal cord antigens, using Western blot analysis (Fig 10A). Data showed that, as compared to control EAE rats, sera from injected EAE rats contained higher concentrations of antibodies directed against a 28 Kda protein, distinct from myelin basic protein (MBP). As myelin oligodendrocyte glycoprotein, a major immunogenic myelin antigen, is a 28 Kda molecule [11], we then performed ELISA experiments to determine the serum concentrations of anti-MOG antibodies (Fig 10B). The concentration of antibodies directed against the MOG peptide 35–55 was greatly increased in injected EAE rats as compared to control EAE rats (84.23+/−22.8 ng/ml vs 20.53+/−4.1 ng/ml in EAE and control rats respectively, p = 0.02, Mann and Whitney test) (Fig 10B). Such an increased antibody response was not observed when considering myelin basic protein (MBP) as a target antigen (Fig 10C). Overall, these data demonstrate that injection of DCs into the CSF of EAE rats induces an exacerbation of clinical signs along with an increased systemic antibody response against MOG.

## Discussion

The use of fluorescent microspheres to track endogeneous APCs has been commonly used to analyze the migratory behavior of DCs in other tissues than the CNS [1], [12]. In our experimental settings, however, one has to consider that microspheres may not have been exclusively taken up by CSF-circulating APCs. Indeed, free microspheres may have penetrated into the inflamed CNS parenchyma before being phagocytized. Nevertheless, several observations which were performed on day 1 post-injection, that is to say an early time point post-injection, argue against this interpretation: i) there were very few free microspheres in the brain of injected EAE rats, ii) microspheres were detected in parenchymal locations that are distant away from the CSF compartments, iii) when injected into the lateral ventricle of normal rats, microspheres did not cross the neuroepithelial layer bordering the lateral ventricle (data not shown). Besides these considerations, it is striking to observe that all the results obtained with the microsphere approach were confirmed, at least qualitatively, when CFSE-labeled DCs were injected into the CSF of EAE rats. It appears to us that quantitative differences rely on the sustained traffic of APCs that likely occurs in the CSF of EAE rats and which cannot be mimicked by a single intra-CSF injection of labeled DCs. However, regarding the presence of microspheres^+^ cells in the CLNs, one cannot exclude the possibility that microspheres have been indeed phagocytized outside the CNS compartments, in the blood or in the CLNs.

The presence of periventricular inflammatory and demyelinating lesions is a full hallmark of MS pathology [13]. We found here that DCs injected into the CSF of EAE rats deeply and specifically penetrated into periventricular demyelinating lesions. Similarly, in EAE rats injected intra-CSF with fluorescent microspheres, periventricular demyelinating lesions contained microspheres^+^/MHC class II^+^ cells, that putatively derived from the CSF. These observations show that, in EAE rats, CSF-circulating DCs are chemoattracted toward such lesions. In all likelihood, these CSF-derived DCs sample antigens locally. However, whether they present antigen to T-cells *in situ* or in the cervical lymph nodes remains to be established. In this regard, it has to be noticed that DCs injected into the brain parenchyma of normal rats migrate little from their site of injection and do not reach the CLNs [10]. This suggests that, similarly, DCs entering the inflamed CNS parenchyma may exert their APC functions *in situ*. Supporting this view, a recent work showed that in EAE mice, blood-derived myeloid DCs infiltrate demyelinating lesions then capture and present myelin antigens *in situ* [6].

Another interesting observation is the particular ability of CSF-circulating DCs to infiltrate the deep penetrating meninges of the inflamed brain. The pia-matter not only covers the hemispheric and cerebellar cortical convolutions, but it lines deep cerebral structures such as the hippocampus or the inner part the brainstem. The pre- and post-capillary vessels are also covered by the pia matter, which thus connects the CSF compartment with the perivascular spaces (Virchow-Robin spaces) [14]. Our data indicate that the pia matter is a major route of traffic for CSF-circulating DCs. In this view, it has to be noticed that meninges have been described as an important site of antigen-presentation and/or B-cell proliferation in EAE or MS [15], [16], [17]. The molecular mechanisms supporting the migration of CSF-DCs along the pia matter have to be clarified. One may hypothesize that chemokines accumulate specifically in the CSF compartment delineated by the penetrating pia-matter. Alternatively, leptomeninengeal cells themselves may secrete chemokines [18]. One may speculate that such a peculiar routage via the pia-matter may allow CSF-DCs: i) to sample soluble antigens that are drained from the interstitial fluid of CNS parenchyma [19], [20], [21], ii) to eventually reach intra-parenchymal locations that are distant away from the ventricles, iii) to target the perivascular spaces of post-capillary venules, where T-cells preferentially infiltrate the CNS [14].

The intra-CSF injection of DCs in EAE rats induced an exacerbation of clinical signs and an increased systemic antibody response against MOG. Interestingly, in different EAE models developed in rats, mice or monkeys, anti-MOG antibodies are considered as highly pathogenic [22], [23], [24], [25]. Similarly, in MS patients, anti-MOG antibodies were evidenced in the serum, CSF and, most importantly, within CNS inflammatory lesions [22], [26]. Based on these results, one may assume that in EAE rats injected with DCs, increased concentrations of serum anti-MOG antibodies may be partly responsible for the aggravation of clinical signs. Moreover, that intra-CSF injected DCs enhanced the systemic antibody response further demonstrates that DCs are able to migrate from the CSF to the CLNs. Interestingly, preliminary data indicate that, in EAE rats, a diversification of the antibody repertoire occurs in the CLNs but not the axillary lymph nodes (data supplement S2). It is thus thinkable that, when targeting the B-cell area of CLNs, DCs injected intra-CSF induce an amplification of the anti-MOG antibody response that develops in the CLNs. Supporting this view, a sub-population of DCs was previously evidenced in the B-cell zone of lymphoid organs [27], [28], [29] and DCs were shown to support the proliferation/differentiation of B-cells [29], [30]. Besides the effects of DCs on B-lymphocytes, it is likely that T-cell driven mechanisms are also triggered by the infiltration of CSF DCs in multiple CNS compartments, as previously shown in a murine model of EAE [6].

In many neuroinflammatory disorders, the intra-CSF accumulation of immune cells is a major diagnosis criterion. Paradoxically, little is know about how and where these CSF-circulating cells migrate, under neuroinflammatory conditions. The present work unravels the complex migratory behavior of CSF-circulating DCs in EAE rats. From the ventricular CSF compartment, DCs have the ability to target the CLNs but also inflammatory lesions that localize in periventricular areas, in the deep penetrating meninges and, to a lesser extend, around parenchymal blood vessels. Our work shows that CSF DCs are thus able to support CNS-targeted immune responses inside as well as outside the inflamed CNS.

## Materials and Methods

### Animals

Animal care and procedures have been conducted according to the guidelines approved by the French regional ethical committee in animal experimentation (CREEA - agreement number 0108) and meet the Neuroscience Society guidelines. Eight to ten weeks old female Dark Agouti rats were obtained from Harlan (Gannat, France).

### Reagents

Murine GM-CSF, human Flt3-L and murine IL-4 were obtained from Peprotech (Tebu). Mouse monoclonal antibodies recognizing rat MHC class II molecules (OX6 antibody), rat CD11b/CD11c (OX42 antibody) or human myelin basic protein (MBP), were purchased from Becton Dickinson Biosciences (Pharmingen). For immuncytochemistry, a fluorescein-conjugated goat anti-mouse antibody (Alexa Fluor 488 or 546, Molecular Probes) was used as a secondary antibody.

### Generation and characterization of rat bone marrow-derived DCs

Rat myeloid DCs were generated from whole bone marrow cultures as previously described [10]. Briefly, rats were sacrificed, and bone marrow was flushed from femurs and tibias using 10 ml of DMEM in a 10 ml syringe with a 26-gauge needle. Bone marrow cells were then re-suspended and passed through a cell strainer (70 µm pore). Cells were washed once in DMEM (Gibco) then cultured in 25 cm^2^ culture plates at a density of 10^6^ cells/ml in IMDM (Gibco) supplemented with 15% FCS (Fetal Clone II, Perbio Science) and antibiotics (penicillin/streptomycin, Invitrogen). Bone marrow cultures were then grown for 7 days at 37°C, 5% CO_2_ in the presence of murine GM-CSF (10 ng/ml) and human Flt-3 ligand (10 ng/ml). By the end of this period, clusters of non adherent cells had formed that were removed, dispersed and re-plated in fresh media consisting of DMEM supplemented with 10% FCS (BioWest), penicillin/streptomycin (Invitrogen), murine GM-CSF (10 ng/ml) and murine IL-4 (10 ng/ml). After 3 days, large numbers of free-floating cells could be harvested, washed once in PBS and used for labeling and *in vivo* experiments. The phenotype and morphology of injected cells was assessed by FACS or immunocytology, as previously described [10] (data supplement S3). Data showed that injected cells expressed a dendritic cell phenotype as demonstrated by the detection of OX42 (CD11b/c), CD11c and MHC class II molecules. Only 25–35% of cells expressed the co-stimulatory molecule CD86, indicating that a great majority of cells displayed an immature DC phenotye. Accordingly, on cytsopin preparations, immunocytological staining of MHC molecules showed that most cells harbored intracytoplasmic MHC class II vesicles, a hallmark of immature DCs (data supplement S3).

### Cell labelling

DCs were labeled with CFSE (carboxyfluorescein diacetate succinimidyl ester, Molecular Probes) as previously described [10]. Briefly, cells were washed once in PBS and incubated for 5 min at 37 °C in 1 µM CFSE. Then, 250 µl of FCS was added and cells were further incubated for 5 min at 37°C before being washed in PBS and re-suspended at a dilution of 3.10^4^ cells/µl in red phenol-free DMEM.

### Experimental allergic encephalomyelitis

Chronic relapsing EAE was induced in a total of 47 female Dark Agouti rats, as previously described [31]. Briefly, 0.5 gram guinea pig spinal cord obtained from Dunkin-Hartley guinea pigs was homogenized with 3 ml saline and the homogenate was then emulsified with 3 ml of Difco's bacto complete Freund's adudjuvant (CFA) H37RA supplemented with 15 mg of Mycobacterium tuberculosis H37RA (Difco Laboratories, Detroit, MI). Rats were injected intradermally into each footpad, under Isoflurane anesthesia, with 0.1 ml of this emulsion. In this EAE model, clinical signs peak on day 12 post-immunization then animals partially recover and experience a second relapse. Rats were weighted and scored daily according to the following clinical scale: 0 = no clinical signs, 1 = tail weakness, 2 = tail paralysis, 3 = incomplete paralysis of one or two hind legs, 4 = complete hind limb paralysis, 5 = moribund

### Stereotaxic injections of fluorescent microspheres

On day 12 post-immunization, EAE rats (n = 14) received an intracerebroventricular (intra-CSF) injection of sterile latex fluorescent microspheres diluted 1/1000. All injections were performed using red-phenol free DMEM as a vehicle. Briefly, each rat was deeply anesthetized by pentobarbital injection, placed in a stereotaxic frame and its head tilted slightly by raising the tooth bar at 5 mm. Ten µl of the solution was then slowly injected in the left lateral ventricle (stereotaxic coordinates: 1,4 mm lateral to the bregma and 4,5 mm down from the surface of the skull) over a period of 3 min, using a Hamilton syringe. The animal remained in the stereotaxic frame with the needle in place thereafter for 1 min and the needle was then slowly removed over a period of 2 min. Following intra-CSF injections, animals were kept in our animal facility before being sacrificed on day 1 post-injection (n = 8), or on day 8 post-injection (n = 6).

### Stereotaxic injections of labeled DCs

Following the same protocol than described above, EAE rats (n = 18) were injected intra-CSF with vehicle (10 µl) containing 3.10^5^ CFSE-labeled DCs. Animals were then sacrificed on day 1 (n = 8) or on day 8 (n = 10) post-injection. In parallel experiments, normal rats (n = 7) were injected intra-CSF with the same preparation of cells and sacrificed on day 1 (n = 4) or 8 (n = 3) post-injection.

### Histological analysis

On day 1 or 8 after intra-CSF injections of DCs or microspheres, animals were anesthetized by halothan inhalation and killed by intraperitoneal injection of an overdose of pentobarbital sodium (100 mg/kg). Brains, cervical lymph nodes and axillary lymph nodes were dissected out and frozen in dry-ice. When needed, frozen tissues were embedded in polyethylene glycol and cut in 14 µm thick sections with a cryostat. After fixation in ethanol, sections were rinsed 3 times in PBS then incubated for 30 min at room temperature with a blocking solution containing 4% bovine serum albumin (Sigma-Aldrich) and 10% normal goat serum. Sections were then incubated overnight at 4°C with mouse monoclonal antibodies directed against CD11b/CD11c (OX42), MHC class II molecules (OX6) or myelin basic protein. OX42 (anti-CD11b/CD11c). All primary antibodies were diluted 1∶100 in blocking solution. After several washes in PBS, sections were incubated for 50 min in blocking solution containing a fluorescein-conjugated goat anti-mouse antibody (dilution 1∶100) then rinsed in PBS and mounted using an aqueous preparation (Fluoroprep, BioMérieux). Images were recorded and analyzed using a computer-assisted system consisting of specific image analysis software (analysis auto, Soft Imaging System GmbH, Munster, Germany), coupled to a light and fluorescent microscope (Zeiss Axioplan II, Oberkochen, Germany) and in some cases, confocal laser scanning microscopy (LSM META Zeiss) was performed to discriminated between phagocytic cells internalization of microspheres and attachment to the cell membrane. For analysis of fluorescence, two views of the same sections were recorded, using appropriate filters, as black and white image, which were then color-coded by software as blue (DAPI, cell nuclei), green (CFSE or cell type markers) or red (fluorescent microspheres or cell type markers). When needed, in order to better visualize red fluorescent microspheres on a black background, red spots were represented as white spots. The number and localization of CFSE^+^ cells was assessed on serial sections of different brain regions (anterior brain, midbrain, brainstem and cerebellum) in EAE rats (n = 8) or in control rats (n = 7). On sections obtained from animals sacrificed on day 1 post-injection (EAE rats: n = 4, normal rats: n = 4), a semi-quantitative analysis was performed to evaluate the presence of CFSE^+^ cells in the following CNS compartments: intraventricular CSF (free-floating or adherent to the choroid plexuses), periventricular parenchyma, superficial meninges, deep-penetrating meninges, perivascular spaces, CNS parenchyma. The following semi-quantitative scale was used: −: none, +/−: occasionnal cells, +: 2 to 5 cells per section, ++: 5 to 10 cells per section, +++: more than 10 cells per section. Also, the injected lateral ventricle was specifically examined and the numbers of intraventricular vs periventricular CFSE^+^ cells were counted in EAE or control rats. Finally, in EAE rats, sections crossing the injected lateral ventricle (3 to 5 sections per animal, n = 4 animals) were immunostained with MBP and the number of CFSE^+^ cells was counted in normal-appearing or demyelinated periventricular white matter. Using a computer-assisted system consisting (analysis auto, Soft Imaging System GmbH, Munster, Germany), we then determined the number of CFSE^+^ cells per mm^2^ surface in demyelinated vs non-demyelinated periventricular white matter.

### Electron microscopy

In some experiments, cervical lymph nodes obtained from control EAE rats (n = 3), EAE rats injected intra-CSF with microspheres (n = 4) or healthy control rats (n = 3) were processed for electron microscopy examination. Briefly, tissues were fixed for 30 min in 2% glutaraldehyde-0.1 M NaCacodylate pH 7.4. They were then washed three times in 0.1 M NaCacodylate/sucrose, pH 7.4 for 15 min and post-fixed with 1% OsO_4_-0.15 M NaCacodylate pH 7.4 for 30 min. After dehydratation in a growing gradient of ethanol, 5 min for each step: 30, 50, 70 and 95%, impregnation steps and inclusion were performed in Epon finally polymerized at 60°C for 48 h. 60–80 nm sections were obtained using an ultramicrotome RMC-MTX (Tucson), and contrasted with uranyl acetate and lead citrate. Observations were performed on a JEOL 1200EX transmission electron microscope (Jeol LTD) equipped with a MegaView II high resolution TEM camera and Analysis Soft Imaging system (Eloïse SARL).

### Flow cytometry

Following intra-CSF injection of CFSE-labeled DCs in normal rats (n = 7) or EAE rats (n = 8), CLNs and axillary lymph nodes were mechanically dissociated, washed once in PBS 1× then filtered through a 70 µm cell strainer (Pharmingen, San Diego, USA). Cells were then fixed in PFA 1% before analysis of CFSE fluorescence. The level of autofluorescence was established on cells obtained from the CLNs or ALNs of non-injected EAE rats or control rats. Alternatively, cells were stained with OX6 antibody (recognizing MHC Class II molecules) as previously described [10]. Briefly, 2 to 5.10^5^ cells were sequentially incubated on ice with a blocking CD16/CD32 monoclonal antibody (Pharmingen, San Diego, USA) and a mouse PE-conjugated OX6 antibody (Pharmingen, San Diego, USA) diluted 1∶50 in PBS containing 2% FCS. After two washes in PBS 1×, cells were then fixed in PFA 1% before being analyzed on a Galaxy flow cytometer (Partec, Germany) with FlowMax software (Partec, Germany).

### Western blot

Western blot experiments were performed to assess the presence of serum antibodies directed against purified myelin basic protein (MBP) (obtained from guinea pig brain, Sigma-Aldrich) or whole spinal cord protein extracts (obtained from normal healthy rats). Purified MBP or pooled spinal cord samples that had been boiled in SDS-PAGE reducing buffer for 5 min at 95°C, were separated on a SDS-PAGE gel and transferred onto a nitrocellulose membrane. The membrane was incubated in PBS containing 0.1% Tween 20 and 5% milk, 1 h at room temperature and incubated with serum samples (dilution 1/10) obtained from control EAE rats (n = 4) or EAE rats injected intra-CSF with DCs (n = 4). Membranes were then washed in PBS containing 0.1% Tween 20 and 1% Milk and incubated with peroxidase-conjugated goat anti-rat IgG immunoglobulin (Jackson, USA) for 1 hour at room temperature. After washing, membranes were incubated with Western Blot Chemoluminescence Reagent (Covalab, France) for 1 min at room temperature and blots were exposed to X-ray films. Semi-quantitative analyses of signals were performed by measuring optical densities with the software Image Quant.

### ELISA

Serum samples were tested by ELISA for the presence of anti-MOG 35–55 antibodies, using a commercially available kit (Anaspec). Assays were performed in duplicate and according to the manufacturer's recommendations. Briefly, 100 µl of undiluted serum samples were incubated in wells of the 96-well microtitration plate pre-coated with MOG 35–55 peptide and a blocking solution. After washing, peroxidase-conjugated antibodies to rat/mouse IgG were added and further incubated for 1 h at room temperature. The reaction was developed after another series of washes by addition of 50 µl of TMB (Tetramethylbenzidine) color substrate solution. Optical densities were then measured on a Multiskan EK ELISA spectrophotometer (Thermo Electron Corporation, Courtaboeuf, France). A calibration curve was established using standard dilutions of mouse anti-MOG IgG and individual values of serum anti-MOG IgGs were then calculated and expressed as ng/ml.

### Statistical analyses

Statistical analyses were performed with the non-parametric Mann and Whitney test. Alternatively, the Student's *t* test was performed when the *n* reached at least 10 in each group and only if values had a Gaussian distribution as assessed by the Shapiro and Wilk test.

## Supporting Information

Data Supplement S1 Microspheres+/MBP+ cells in a periventricular demyelinating lesion. EAE rats were injected intra-CSF with fluorescent microspheres (red in A and D, white in C) and were then sacrificed on day 8 post-injection. Brain sections were immunostained with an anti-MBP antibody in order to visualize myelin (green in A and D, white in B). Microphotograph in A shows that a large demyelinated area adjacent to the third ventricle, is filled with microspheres+ cells. Within this large demyelinated area, a partially demyelinated area (solid square) contains micropheres+/MBP+ cells. Higher magnification views of this area are shown in B (MBP staining), C (microspheres) and D (merge). V3: third ventricle. Scale bars: 100 mm (A), 10 mm (B–D)
(0.92 MB TIF)

## References

[1] Matsuno K, Ezaki T, Kudo S, Uehara Y (1996). A life stage of particle-laden rat dendritic cells in vivo: their terminal division, active phagocytosis, and translocation from the liver to the draining lymph.. J Exp Med.

[2] Liu YJ (2001). Dendritic cell subsets and lineages, and their functions in innate and adaptive immunity.. Cell.

[3] Pashenkov M, Huang YM, Kostulas V, Haglund M, Soderstrom M (2001). Two subsets of dendritic cells are present in human cerebrospinal fluid.. Brain.

[4] Pashenkov M, Teleshova N, Kouwenhoven M, Smirnova T, Jin YP (2002). Recruitment of dendritic cells to the cerebrospinal fluid in bacterial neuroinfections.. J Neuroimmunol.

[5] Serafini B, Rosicarelli B, Magliozzi R, Stigliano E, Capello E (2006). Dendritic cells in multiple sclerosis lesions: maturation stage, myelin uptake, and interaction with proliferating T cells.. J Neuropathol Exp Neurol.

[6] Bailey SL, Schreiner B, McMahon EJ, Miller SD (2007). CNS myeloid DCs presenting endogenous myelin peptides ‘preferentially’ polarize CD4+ T(H)-17 cells in relapsing EAE.. Nat Immunol.

[7] Kostulas N, Li HL, Xiao BG, Huang YM, Kostulas V (2002). Dendritic cells are present in ischemic brain after permanent middle cerebral artery occlusion in the rat.. Stroke.

[8] Carson MJ, Reilly CR, Sutcliffe JG, Lo D (1999). Disproportionate recruitment of CD8+ T cells into the central nervous system by professional antigen-presenting cells.. Am J Pathol.

[9] Karman J, Ling C, Sandor M, Fabry Z (2004). Initiation of immune responses in brain is promoted by local dendritic cells.. J Immunol.

[10] Hatterer E, Davoust N, Didier-Bazes M, Vuaillat C, Malcus C (2006). How to drain without lymphatics? Dendritic cells migrate from the cerebrospinal fluid to the B-cell follicles of cervical lymph nodes.. Blood.

[11] Amiguet P, Gardinier MV, Zanetta JP, Matthieu JM (1992). Purification and partial structural and functional characterization of mouse myelin/oligodendrocyte glycoprotein.. J Neurochem.

[12] Mitra R, Dharajiya N, Kumari L, Varalakshmi C, Khar A (2004). Migration of antigen presenting cells from periphery to the peritoneum during an inflammatory response: role of chemokines and cytokines.. Faseb J.

[13] Newcombe J, Hawkins CP, Henderson CL, Patel HA, Woodroofe MN (1991). Histopathology of multiple sclerosis lesions detected by magnetic resonance imaging in unfixed postmortem central nervous system tissue.. Brain.

[14] Bechmann I, Galea I, Perry VH (2007). What is the blood-brain barrier (not)?. Trends Immunol.

[15] Serafini B, Rosicarelli B, Magliozzi R, Stigliano E, Aloisi F (2004). Detection of ectopic B-cell follicles with germinal centers in the meninges of patients with secondary progressive multiple sclerosis.. Brain Pathol.

[16] Magliozzi R, Howell O, Vora A, Serafini B, Nicholas R (2007). Meningeal B-cell follicles in secondary progressive multiple sclerosis associate with early onset of disease and severe cortical pathology.. Brain.

[17] Shin T, Kojima T, Tanuma N, Ishihara Y, Matsumoto Y (1995). The subarachnoid space as a site for precursor T cell proliferation and effector T cell selection in experimental autoimmune encephalomyelitis.. J Neuroimmunol.

[18] Christodoulides M, Makepeace BL, Partridge KA, Kaur D, Fowler MI (2002). Interaction of Neisseria meningitidis with human meningeal cells induces the secretion of a distinct group of chemotactic, proinflammatory, and growth-factor cytokines.. Infect Immun.

[19] Weller RO (1998). Pathology of cerebrospinal fluid and interstitial fluid of the CNS: significance for Alzheimer disease, prion disorders and multiple sclerosis.. J Neuropathol Exp Neurol.

[20] Schley D, Carare-Nnadi R, Please CP, Perry VH, Weller RO (2006). Mechanisms to explain the reverse perivascular transport of solutes out of the brain.. J Theor Biol.

[21] Carare RO, Bernardes-Silva M, Newman TA, Page AM, Nicoll JA (2008). Solutes, but not cells, drain from the brain parenchyma along basement membranes of capillaries and arteries: significance for cerebral amyloid angiopathy and neuroimmunology.. Neuropathol Appl Neurobiol.

[22] Genain CP, Cannella B, Hauser SL, Raine CS (1999). Identification of autoantibodies associated with myelin damage in multiple sclerosis.. Nat Med.

[23] Marta CB, Oliver AR, Sweet RA, Pfeiffer SE, Ruddle NH (2005). Pathogenic myelin oligodendrocyte glycoprotein antibodies recognize glycosylated epitopes and perturb oligodendrocyte physiology.. Proc Natl Acad Sci U S A.

[24] Jagessar SA, Smith PA, Blezer E, Delarasse C, Pham-Dinh D (2008). Autoimmunity against myelin oligodendrocyte glycoprotein is dispensable for the initiation although essential for the progression of chronic encephalomyelitis in common marmosets.. J Neuropathol Exp Neurol.

[25] Linington C, Bradl M, Lassmann H, Brunner C, Vass K (1988). Augmentation of demyelination in rat acute allergic encephalomyelitis by circulating mouse monoclonal antibodies directed against a myelin/oligodendrocyte glycoprotein.. Am J Pathol.

[26] O'Connor KC, Appel H, Bregoli L, Call ME, Catz I (2005). Antibodies from inflamed central nervous system tissue recognize myelin oligodendrocyte glycoprotein.. J Immunol.

[27] Lindquist RL, Shakhar G, Dudziak D, Wardemann H, Eisenreich T (2004). Visualizing dendritic cell networks in vivo.. Nat Immunol.

[28] Grouard G, Durand I, Filgueira L, Banchereau J, Liu YJ (1996). Dendritic cells capable of stimulating T cells in germinal centres.. Nature.

[29] Balazs M, Martin F, Zhou T, Kearney J (2002). Blood dendritic cells interact with splenic marginal zone B cells to initiate T-independent immune responses.. Immunity.

[30] Dubois B, Barthelemy C, Durand I, Liu YJ, Caux C (1999). Toward a role of dendritic cells in the germinal center reaction: triggering of B cell proliferation and isotype switching.. J Immunol.

[31] Fernandez M, Giuliani A, Pirondi S, D'Intino G, Giardino L (2004). Thyroid hormone administration enhances remyelination in chronic demyelinating inflammatory disease.. Proc Natl Acad Sci U S A.

