# Supplementary figures and images for: Cerebrospinal Fluid Dendritic Cells Infiltrate the Brain Parenchyma and Target the Cervical Lymph Nodes under Neuroinflammatory Conditions
Source: PLoS One. 2008 Oct 2;3(10):e3321. doi: 10.1371/journal.pone.0003321 (PMC2552991; doi:10.1371/journal.pone.0003321)

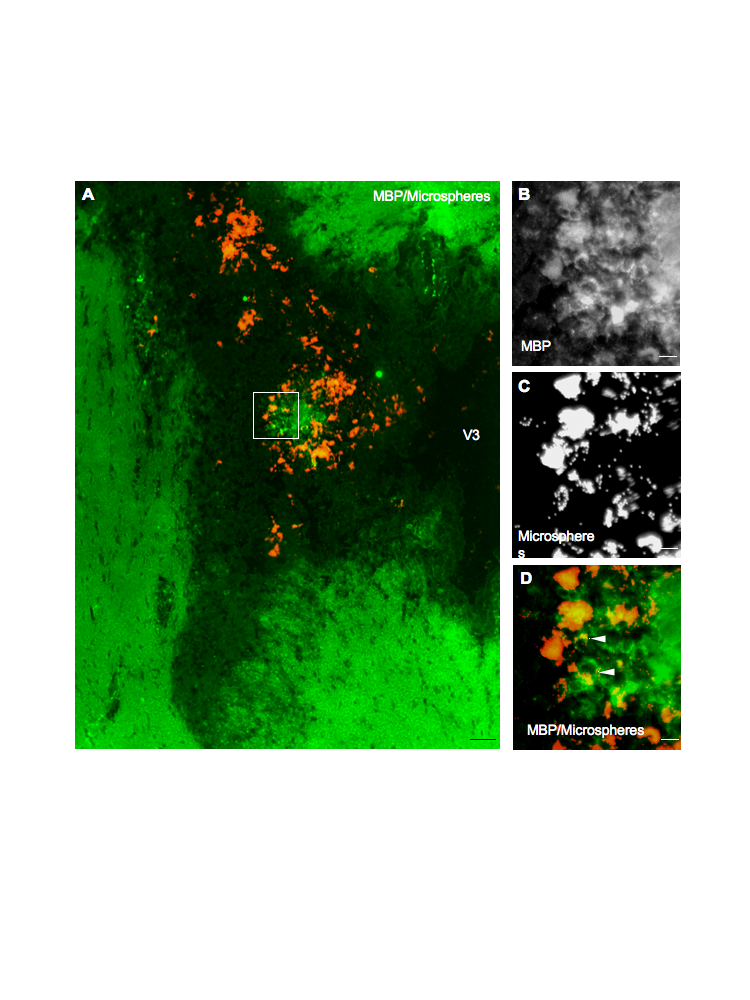

Supplement: Data Supplement S1 — Microspheres+/MBP+ cells in a periventricular demyelinating lesion. EAE rats were injected intra-CSF with fluorescent microspheres (red in A and D, white in C) and were then sacrificed on day 8 post-injection. Brain sections were immunostained with an anti-MBP antibody in order to visualize myelin (green in A and D, white in B). Microphotograph in A shows that a large demyelinated area adjacent to the third ventricle, is filled with microspheres+ cells. Within this large demyelinated area, a partially demyelinated area (solid square) contains micropheres+/MBP+ cells. Higher magnification views of this area are shown in B (MBP staining), C (microspheres) and D (merge). V3: third ventricle. Scale bars: 100 mm (A), 10 mm (B–D) (0.92 MB TIF) [file pone.0003321.s001.tif]
